# Urbanization and mental health

**DOI:** 10.4103/0972-6748.64028

**Published:** 2009

**Authors:** Kalpana Srivastava

**Affiliations:** Editor, IPJ E-mail: kalpanasrivastava@hotmail.com

Urbanization, defined as the increase in the number of cities and urban population, is not only a demographic movement but also includes, social, economic and psychological changes that constitute the demographic movement. It is a process that leads to the growth of cities due to industrialization and economic development (M. Tayfun Turan, Aslı Besirl 2008). The rapid increase in urban population worldwide is one among the important global health issues of the 21^st^century. According to the projections of the United Nations Population Division, by 2030, more people in the developing world will live in urban than rural areas; by 2050, two-thirds of its population is likely to be urban. The scenario in India is also affected by this trend. In India approximately 28% of the India’s population lives in cities and this is expected to increase to 41% by the year 2020 (UN World Urbanization Prospects 2008).

Urbanization brings with it a unique set of advantages and disadvantages. This demographic transition is accompanied by economic growth and industrialization, and by profound changes in social organization and in the pattern of family life. Urbanization affects mental health through the influence of increased stressors and factors such as overcrowded and polluted environment, high levels of violence, and reduced social support.

Movement of population to urban areas has led to large number of older men and women left to look after themselves in the rural areas, while the young generation lives in the cities for livelihood. This also leads to less availability of caregivers for old people. It is worth mentioning here that by 1990, majority (58%) of the world’s population aged 60 years and over was already found to be living in developing countries. By 2020, this proportion would have risen to 67%. Over this period of 30 years, this oldest sector of the population would have increased in number by 200% in developing countries as compared to 68% in the developed world (Anon 1994, Murray CJ, Lopez AD 1997).

Impact of urbanization is associated with an increase in mental disorders. The reason is that movement of people to urban area needs more facilities to be made available and infrastructure to grow. This does not happen in alignment with the increase of population Hence, lack of adequate infrastructure increases the risk of poverty and exposure to environmental adversities. Further this also decreases social support (Desjarlais *et al*., 1995) as the nuclear families increase in number. Poor people experience environmental and psychological adversity that increases their vulnerability to mental disorders (Patel, 2001).

A report by World Health Organization (WHO) (World Health Organization) has enumerated that mental disorders account for nearly 12% of the global burden of disease. By 2020, these will account for nearly 15% of disability-adjusted life-years (DALYs) lost to illness. Incidentally, the burden of mental disorders is maximal in young adults, which is considered to be the most productive age of the population. Developing countries are likely to see a disproportionately large increase in the burden attributable to mental disorders in the coming decades (WHO Mental Health Context 2003).

The range of disorders and deviancies associated with urbanization is enormous. Some of the disorders are severe mental disorders, depression, substance abuse, alcoholism, crime, family disintegration, and alienation. Dementia and major depression are two, dementia and major depression are the two leading contributors, accounting, respectively, for one-quarter and one-sixth of all disability adjusted life years (DALYs) in this group. Most people with dementia live in developing countries: 60% in 2001 is projected to rise up to 71% by 2040. Rates of increase are not uniform: numbers are forecast to increase by 100% in developed countries between 2001 and 2040, but by more than 300% in India, China, and their South Asian and Western Pacific neighbors (Trivedi 2008).

When we refer to psychiatric disorders anxiety and depression are more prevalent among urban women than men and, are believed to be more prevalent in poor than in non-poor urban neighborhoods (Naomar Almeida-Filho *et al* 2004). The meta analysis by Reddy and Chandrashekhar(1998) revealed higher prevalence of mental disorders in urban area i.e., 80.6%, whereas it was 48.9% in rural area. Mental disorders primarily composed of depression and neurotic disorders.

Socioeconomic stress is considered to be affecting mental health of women. Results of randomized control trials involving individual or group counseling sessions led by community health workers or nurses, either as the principal intervention or in combination with inexpensive drug therapies have indicated the role of counseling intervention among women (Ricardo Araya *et al*., 2007,Vikram Patel *et al*., 2003). Increase of nuclear families in urban society has led to increase in cases of violence against women in general. Among them, intimate-partner violence links to alcohol abuse and women’s mental health. Analysis of community-based data from eight urban areas in the developing world indicates that mental and physical abuse of women by their partners is distressingly common with negative consequences for women’s physical and psychological well being (Lori L. Heise *et al* 1994). Poverty and mental health have a complex and multidimensional relationship. The urbanization leads to forming set of group as “fringe population” who earn on daily basis (Mursaleena Islam etal 2006). An Indian study in a slum community north of Mumbai indicates high incidence of alcoholism among men and verbal abuse of women by their husbands (Shubhangi R. Parkar, Johnson Fernandes, and Mitchell G. Weiss 2003). The WHO analysis also documented a close association between the experience of violence and women’s mental health(2005). Women are particularly vulnerable and they often disproportionately bear the burden of changes associated with urbanization. Domestic violence is also highly prevalent in urban areas. In both developed and developing countries, women living in urban settings are at greatest risk to be assaulted by their intimates (Kessler RC, Sonnega A, Bromet E, Hughes M, Nelson CB (1995).

The model of cultural transformation especially from rural to modern society, is considered to be one of the reasons of psychological disorder. However stress caused by transition from rural culture to urban culture cannot be denied as one of the factors leading to stress-related problems. Cultural factors interplay with urban dynamics in a unique manner. Understanding how cultural dynamics articulate with adaptation to urban life may facilitate proper management of mental disorders in cities. In the assessment and treatment of patients living in urban areas, contextual cultural factors also play an important role (Caracci G, Mezzich JE 2001).

There is a need to create awareness about mental illness across all sections of the society. Urbanization is thus seen as a natural corollary of growth. Awareness about its impact on health and more so on mental health will act as a facilitator of change in growing Indian economy.

## References

[CIT1] Anon (1994) (1994). Assessment of mortality and disability from diseases, injuries and risk factors in 1990 and projected to 2020. Editorial: City Limits. In The Lancet Vol.

[CIT2] Caracci G, Mezzich JE (2001). Culture and urban mental health. Psychiatr Clin North Am.

[CIT3] Kessler RC, Sonnega A, Bromet E, Hughes M, Nelson CB Post-traumatic stress disorder in the National Co-morbidity Survey. Arch Gen Psychiatry.

[CIT4] Lori L, Heise (1994). “Violence Against Women: A Neglected Public Health Issue in Less Developed Countries,”. Social Science and Medicine.

[CIT5] Tayfun Turan M, Aslı Besiril (2008). Impacts of urbanization process on men. Anatolian Journal of Psychiatry.

[CIT6] Mursaleena Islam, Mark R, Montgomery, Shivani Taneja (2006). Urban Health and Care-Seeking Behavior: A Case Study of Slums in India and the Philippines (Bethesda, MD: PHRPlus Program, Abt Associates.

[CIT7] Murray CJ, Lopez AD (1997). Alternative projections of mortality and disability by cause 1990-2020: Global burden of disease study. Lancet.

[CIT8] Naomar Almeida-Filho (2004). “Social Inequality and Depressive Disorders in Bahia, Brazil: Interactions of Gender, Ethnicity, and Social Class,”. Social Science and Medicine.

[CIT9] Patel V, Leon D, Walt G (2001). Poverty, inequality, and mental health in developing countries. Poverty, inequality and health: an international perspective.

[CIT10] Reddy M.V, Chandrashekhar C. R (1998). Prevalence of Mental and behavioral disorders in India:a meta- analysis. Indian Journal of Psychiatry.

[CIT11] Ricardo Araya (2007). Treating Depression in Primary Care in Low-Income Women in Santiago, Chile: A Randomized Controlled Trial,”. Lancet.

[CIT12] Shubhangi R. Parkar, Johnson Fernandes, Mitchell G. Weiss (2003). “Contextualizing Mental Health: Gendered Experiences in a Mumbai Slum,”. Anthropology and Medicine.

[CIT13] Sunita Kishor, Kiersten Johnson (2004). Profiling Domestic Violence: A Multi-Country Study (Calverton, MD: Measure DHS and ORC/Macro).

[CIT14] Trivedi Jitendra, Sareen Himanshu, Dhyani Dhyani, Mohan Mohan (2008). Rapid urbanization - Its impact on mental health. A South Asian region perspective Indian Journal of Psychiatry.

[CIT15] (2008). UN, World Urbanization Prospects The 2007 Revision; and Mark R. Montgomery, “The Urban Transformation of the Developing World,”. Science.

[CIT16] Vikram Patel (2003). “Treatment and Prevention of Mental Disorders in Low-Income and Middle-Income Countries,”. Lancet.

[CIT17] (2003). World Health Organization. The Mental Health Context, Mental Health Policy and Service Guidance Package ISBN 92 4 154594 1(NLM classification: WM 30).

[CIT18] (2005). World Health Organization, WHO Multi-Country Study on Women’s Health and Domestic Violence Against Women: Summary Report of Initial Results on Prevalence, Health Outcomes and Women’s Responses.

